# Léiomyome vésical entraînant la destruction d'un rein

**DOI:** 10.11604/pamj.2016.24.10.6846

**Published:** 2016-05-04

**Authors:** Mehdi Kehila, Karima Mekni, Hassine Saber Abouda, Maher Chtourou, Dorra Zeghal, Mohamed Badis Chanoufi

**Affiliations:** 1 Service C du Centre de Maternité et de Néonatologie de Tunis, Faculté de Médecine de Tunis, Université de Tunis El Manar, Tunisie; 2 Service d'Urologie, Hôpital Habib Thameur, Faculté de Médecine de Tunis, Tunisie

**Keywords:** Vessie, cystoscopie, léiomyome, tumeur, insuffisance rénale, Bladder, cystoscopy, leiomyoma, tumour, renal failure

## Abstract

Le léiomyome de la vessie est une tumeur bénigne rare réputée avoir un bon pronostic après traitement chirurgical. Ceci n'est malheureusement pas toujours vrai. Nous rapportons le cas d'une patiente âgée de 33 ans qui a consulté pour des douleurs lombaires droites. Les explorations réalisées ont conclu à une tumeur solide du plancher vésical avec, en amont, un rein droit non fonctionnel et des voies urinaires gauches dilatées. La cystoscopie a objectivé une tumeur solide de la vessie périméatique droite. Des biopsies tumorales ont été faites en même temps qu'une montée de sonde double J gauche. L’étude anatomopathologique a conclu à un léiomyome vésical. Elle a eu une myomectomie par voie transvésicale. Les suites opératoires étaient simples. La patiente a toutefois gardé comme séquelle un rein totalement détruit.

## Abstract (en)

Leiomyoma of the bladder is a rare benign tumor deemed to have a good prognosis after surgical treatment. This is unfortunately not always true. We report the case of a 33 year-old patient who consulted for lumbar pain on right side. Exploration of patient revealed bladder floor solid tumor with non-functioning right kidney and left urinary tract dilation. Cystoscopy objectified solid tumor of the right perimeatal bladder. Tumor biopsies were performed together with the insertion of a left double J stent. Anatomo-pathologic study showed leiomyoma of the bladder. The patient underwent laparoscopic myomectomy. The postoperative course was uneventful. Pathological effect and sequelae was complete distruction of kidney.

## Introduction

Les tumeurs mésenchymateuses bénignes de la vessie sont rares, occupant moins de 0,43% des tumeurs vésicales [1]. Le léiomyome est la forme histologique la plus fréquente. Il représente 35% des tumeurs bénignes avec une prédominance féminine [2–4]. Le diagnostic repose sur des critères de présomption cliniques et radiologiques et la confirmation est histologique [5]. Après exérèse, son pronostic est classiquement favorable [3]. Nous rapportons un cas de léiomyome vésical révélé par des douleurs lombaires droites.

## Patient et observation

**Figure 1:**
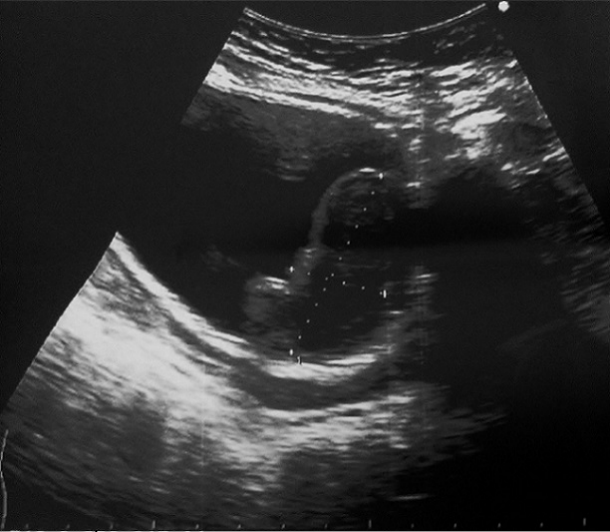
Échographie par voie sus-pubienne montrant une masse tissulaire hétérogène, polylobée, à développement endovésical

Il s'agit d'une patiente âgée de 33 ans, sans antécédent pathologique notable, qui a consulté son médecin traitant pour des douleurs lombaires droites évoluant depuis 2 semaines. Ces douleurs étaient d'aggravation progressive associées à des signes urinaires à type de pollakiurie et dysurie sans hématurie. L'examen clinique et une échographie abdomino-pelvienne réalisées ont révélé une tumeur pelvienne située entre la vessie et le vagin avec une dilatation urétéro-pyélocalicielle bilatérale. Suspectant devant ce tableau un cancer du col engainant les uretères, la patiente a été adressée au service de gynécologie. L'examen a trouvé une patiente en bon état général. L'examen abdomino-pelvien a trouvé une fosse lombaire droite sensible. Au speculum, le col était macroscopiquement sain. Au toucher vaginal, on palpait une masse bombant le tiers supérieur de la face antérieure du vagin sans l'envahir avec une muqueuse vaginale en regard lisse et souple. Cette tumeur était régulière, arrondie, de consistance ferme, non douloureuse, rappelant un léiomyome utérin. L’échographie réalisée par voie sus-pubienne et endo-vaginale a mis en évidence une masse tissulaire hétérogène, polylobée, à développement endovésical, située en regard de la partie supérieure de la paroi vaginale antérieure et mesurant 90*67*34 mm (Figure 1). Cette tumeur paraissait être indépendante de l'utérus.

**Figure 2:**
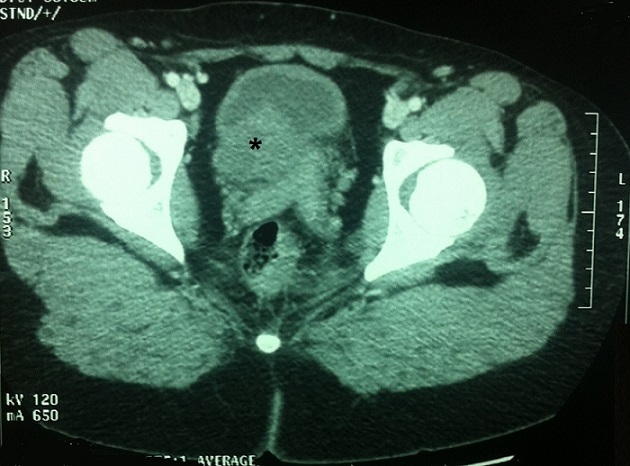
Coupe TDM montrant une masse tissulaire du plancher vésical à extension pelvienne droite comprimant le méat urétéral droit

La fonction rénale était normale. Un uro-scanner réalisé a conclu à une masse tissulaire du plancher vésical à extension pelvienne droite envahissant l'uretère pelvien droit ainsi que son méat. La masse semblait envahir partiellement le méat uretèro-vésical gauche. Elle s'adossait contre la région utérine cervico-isthmique dont une éventuelle extension ne pouvait être éliminée. Le rein droit était non fonctionnel avec une absence totale de sécrétion. A gauche, le rein était de fonction normale, siège d'une dilatation urétéro-pyélo-calicielle modérée. Il n'existait pas de signes radiologiques en faveur d'une extension vers les organes de voisinage ni à distance (Figure 2).

**Figure 3:**
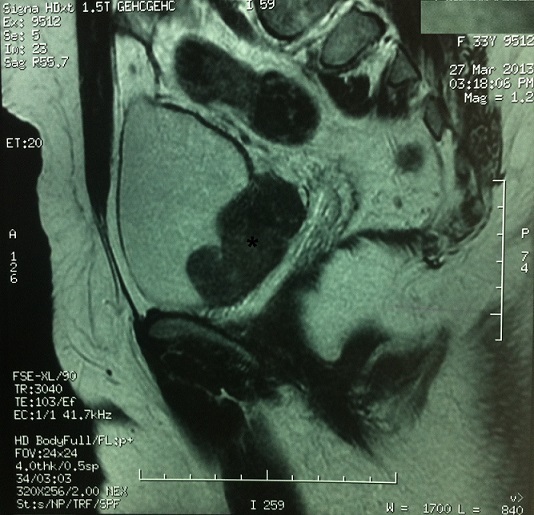
Coupe IRM montrant un processus tissulaire bourgeonnant de la paroi postéro-latérale droite de la vessie à extension extra-vésicale. La tumeur est en discret hyposignal T2. Elle vient en contact étroit avec le vagin dont elle est séparée par un liseré de sécurité

Devant le doute sur la nature de la tumeur, on a complété par une Uro-IRM qui a conclu à un processus tissulaire bourgeonnant de la paroi postéro-latérale droite de la vessie engainant l'uretère homolatéral avec extension extra-vésicale postéro latérale et infiltration de la graisse de voisinage. La tumeur était en discret hyposignal T2 et T1 par rapport au muscle. Le processus tumoral se rehaussait après injection de gadolinium mais il existait une zone d′hyposignal pouvant correspondre à de la nécrose. Il était en contact étroit avec le vagin dont il était séparé par un liseré de sécurité (Figure 3).

**Figure 4:**
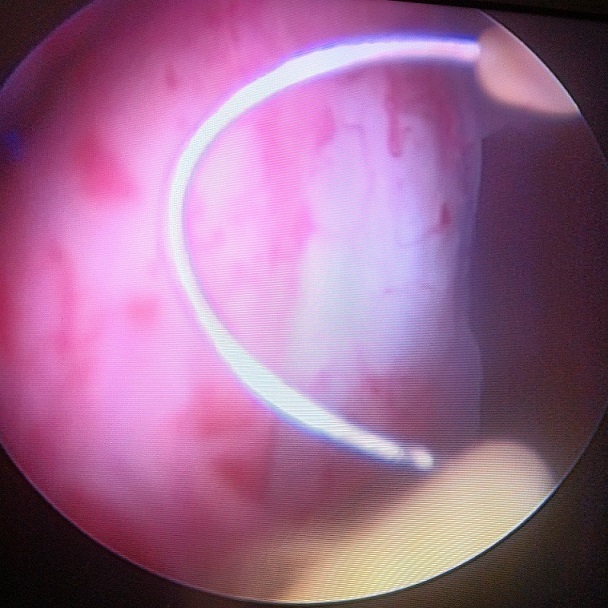
Cystoscopie montrant une tumeur bourgeonnante de 5 cm, trigonale, latéralisée à droite recouverte par une muqueuse vésicale saine

Devant les conclusions des deux examens d'imagerie une tumeur vésicale maligne a été évoquée. Une cystoscopie avec biopsies a été indiquée. En cystoscopie, il y avait une tumeur bourgeonnante de 5 cm, trigonale, latéralisée à droite recouverte par une muqueuse vésicale strictement normale (Figure 4).

**Figure 5:**
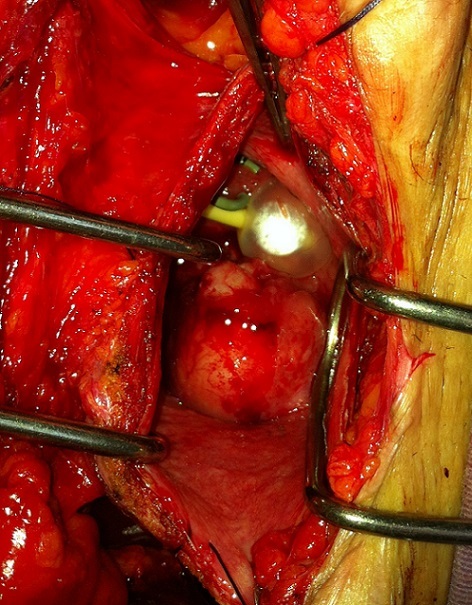
Ouverture de la vessie mettant en évidence la tumeur développée au niveau du trigone vésical. Elle est latéralisée à droite obstruant le méat urétéral droit. Le méat urétéral gauche est cathétérisé par la sonde double J

Le méat droit était soulevé par la tumeur ce qui le rendait difficilement identifiable. Le méat gauche était bien visible soulevé aussi partiellement par la tumeur. Une montée de sonde double J à gauche a été réalisée afin de préserver le rein. Une résection endoscopique de la tumeur a été réalisée à visée biopsique vu la taille importante de la tumeur rendant une résection complète techniquement difficile. La biopsie a ramené des copaux rappelant ceux qu'on résèque lors des myomectomies faites par hystéroscopie ou ceux d'un adénome de la prostate. L'examen anatomo-pathologique a montré une prolifération de cellules fusiformes à différenciation musculaire lisse, disposées en faisceaux longs et entrecroisés signant un léiomyome vésical. Une myomectomie transvésicale a été indiquée devant la taille importante de la tumeur. La voie d'abord était de type Pfannenstiel. La vessie a été ouverte au niveau de sa face antérieure, mettant en évidence la tumeur (Figure 5).

**Figure 6:**
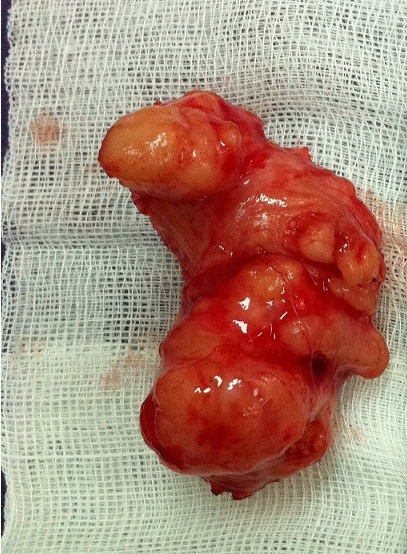
Léiomyome vésical après énucléation mesurant 90x6x30 mm

Celle-ci était polylobée, solide de 5 cm de grand axe vascularisée se développant au niveau du trigone vésical, en sous muqueux. Elle était latéralisée à droite obstruant le méat urétéral droit. Le méat urétéral gauche était cathétérisé par la sonde double J (Figure 5). La résection de la tumeur était faite en monobloc par énucléation (Figure 6). Les suites opératoires ont été simples. L’étude anatomopathologique de la pièce opératoire a confirmé le diagnostic de léiomyome vésical. La sonde double J a été enlevée après deux mois. La patiente a gardé une fonction rénale correcte. Le contrôle échographique a montré une disparition totale de la dilatation de la voie excrétrice gauche objectivée en pré-opératoire. Le suivi de la patiente a consisté en un contrôle clinique, un dosage de la créatininémie et une échographie rénale tous les 6 mois. Après trois ans de recul, le contrôle clinique, biologique et radiologique est resté inchangé avec une patiente asymptomatique, une fonction rénale normale et des cavités excrétrices gauches non dilatées.

## Discussion

Les léiomyomes des voies urinaires sont des tumeurs bénignes rares avec une proportion qui varie de 0,5 à 5% des tumeurs primitives des voies excrétrices [2–7]. Le léiomyome peut se développer en endovésical, intra-mural, ou dans un endroit extra vésical (bassinet, uretère et urètre) [8]. La localisation vésicale constitue la localisation la plus fréquente et représente 35% des tumeurs bénignes vésicales [2, 5, 7]. Plusieurs études rapportent une prédominance féminine avec un sexe ratio de 3 femmes pour un homme [3, 4, 9–11]. L’étiopathogénie de cette tumeur reste mal connue [5, 7]. Cependant, une implication hormonale est suggérée [12]. La présentation clinique peut être extrêmement polymorphe, elle dépend de la taille ainsi que de l′emplacement de la tumeur [5–7]. Une symptomatologie bruyante se voit surtout dans les formes à développement intra vésical (pollakiurie, dysurie, douleurs lombaires, infection urinaire ou plus rarement hématurie) [3, 5]. Alors que dans les formes à développement extra vésical ou intra-mural, la symptomatologie clinique est pauvre et tardive, liée à la compression extrinsèque des structures de voisinage [6]. Dans le cas de notre patiente, le léiomyome a entrainé la destruction du rein droit par obstruction du méat urétéral et une dilatation urétéro-pyélo-calicielle gauche. Ce retentissement en amont se voit habituellement dans les tumeurs infiltrant le muscle vésical. Cependant, la dilatation dans le cas de notre patiente, est plutôt due à la localisation périméatique droite de la tumeur expliquant la destruction totale du rein droit par obstruction extrinsèque de la portion intramurale de l'uretère. A gauche, le soulèvement du trigone par la tumeur a déformé le trajet de l'uretère terminal rappelant l'aspect en hameçon de celui-ci observé dans le cas d'adénomes de prostate obstructifs. En plus d'un examen clinique minutieux pouvant déjà orienter vers l'origine et la nature de la tumeur, l’échographie, l'IRM et la cystoscopie sont des outils utiles pour faire un diagnostic précis [2].

Les examens d'imagerie en préopératoire sont particulièrement utiles pour confirmer l'origine vésicale de la tumeur pour éviter de fistuliser une tumeur digestive ou gynécologique dans la vessie lors de la résection transurétrale. L’échographie pelvienne montre classiquement une masse solide, homogène, échogène, recouverte par une muqueuse intacte plus échogène que la tumeur. Dans le cas de notre patiente la tumeur était plutôt hétérogène. L’échographie par voie endovaginale semble aussi intéressante permettant de distinguer la muqueuse du reste de la masse [5, 6]. En tomodensitométrie, la tumeur apparaît comme une masse tissulaire homogène peu rehaussée par le produit de contraste, bien limitée, localisée le plus souvent au niveau du trigone et du plancher vésical avec un effet de masse sur les organes adjacents sans signe d'envahissement ou d'infiltration tumorale [3, 5, 6].

Comme tout examen complémentaire, l'uro-scanner a ses faux positifs. En effet, chez notre patiente un envahissement de l'uretère droit et de son méat a été signalé nous orientant vers le diagnostic de malignité. En IRM, le léiomyome vésical est le plus souvent homogène de contours réguliers, de signal intermédiaire ou en hyposignal en séquence pondérée (Sp) T1 et en hyposignal en Sp T2, comparable au léiomyome de l'utérus [13]. Dans notre cas il était en hyposignal T1 et T2. Il peut parfois être hétérogène avec des zones de signal élevé correspondant à la dégénérescence mucoïde ou fibroyaline [5, 9]. Le diagnostic différentiel, en imagerie, peut se poser avec l'hémangiome, le phéochromocytome et surtout le léiomyosarcome vésical [5]. Dans notre cas, même si des critères de bénignité étaient retrouvés à l'examen clinique, les constatations radiologiques (notamment l'infiltration de la graisse périvésicale) nous ont orientés à tort vers le diagnostic de malignité. En cystoscopie, la tumeur apparaît classiquement comme un bombement intravésical. La muqueuse vésicale recouvrant la tumeur dans le cas de notre patiente était régulière et d'aspect macroscopiquement normal en faveur de la bénignité de la tumeur. Ceci étant l'aspect le plus fréquemment rapporté dans la littérature [3]. Certains auteurs ont décrit des muqueuses ulcérées posant le diagnostic différentiel avec une tumeur maligne [3]. Le diagnostic de certitude reste histologique, le léiomyome apparaît comme une tumeur conjonctive bien limitée, de couleur blanc nacrée, constituée d'une prolifération d'architecture fasciculée, faite de faisceaux de cellules fusiformes, entrecroisées, développées aux dépens de la musculeuse de la paroi vésicale, sans atypie nucléaire ni mitose. Cette tumeur est recouverte d'une muqueuse vésicale saine, rarement oedématiée ou érodée [3, 5]. Le traitement est chirurgical: cystectomie partielle avec ou sans réimplantation urétérale. Dans la variété sous muqueuse certains auteurs proposent l’énucléation (ce qui a été fait pour notre patiente) ou la résection endoscopique sous réserve d'une petite taille [2, 3, 13]. Ainsi, le traitement des léiomyomes est déterminé principalement par leur taille et leur emplacement anatomique. Dans le cas de notre patiente, nous avons opté pour l’énucléation chirurgicale devant la taille de la tumeur et surtout son retentissement en amont. Certains auteurs proposent l'abstention thérapeutique avec surveillance, devant les léiomyomes de volume stable, asymptomatique et sans aucune répercussion sur l'appareil urinaire [4].

Le pronostic est classiquement favorable après exérèse. Aucun cas de dégénérescence maligne n'a été rapporté [3, 4]. Malheureusement, dans le cas de notre patiente, le léiomyome a entrainé une destruction totale d'un rein. C'est dire qu'il faut être très vigilent face à ce type de tumeur qui même si réputée de bon pronostic, peut être à l'origine de complications gravissimes.

## Conclusion

Le léiomyome vésical est une tumeur très rare. Il faut savoir l’évoquer devant toute tumeur vésicale d'allure bénigne. La nature et la gravité du tableau clinique dépendent essentiellement de l'emplacement de la tumeur plutôt que de sa taille. L'IRM est actuellement l'examen de choix pour le diagnostic et l’étude de ses rapports avec les organes de voisinage. Le diagnostic de certitude est histologique, généralement établi grâce à des biopsies par voie cystoscopique. Son traitement est chirurgical. Le pronostic à long terme est excellent en dehors des séquelles rénales déjà causées avant le traitement et qui sont parfois irréversibles.
